# Impacts of tacrolimus and glucocorticoids on peripheral blood T and B lymphocyte subsets in myasthenia gravis

**DOI:** 10.3389/fimmu.2025.1667799

**Published:** 2025-10-15

**Authors:** Xuan Wu, Wei Chen, Huanhuan Song, Guorong Xu

**Affiliations:** ^1^ Department of Neurology, The First Affiliated Hospital of Fujian Medical University, Fuzhou, China; ^2^ Department of Neurology, National Regional Medical Center, Binhai Campus of the First Affiliated Hospital, Fujian Medical University, Fuzhou, China; ^3^ Department of Neurology and Institute of Neurology of First Affiliated Hospital, Institute of Neuroscience, Fujian Medical University, Fuzhou, China

**Keywords:** myasthenia gravis, tacrolimus, glucocorticoids, lymphocytes, T lymphocytes, B lymphocytes

## Abstract

**Background:**

This study aimed to investigate the impact of tacrolimus on peripheral T and B lymphocyte subsets in myasthenia gravis (MG) patients compared to glucocorticoid treatment.

**Methods:**

This study retrospectively included MG patients at the First Affiliated Hospital of Fujian Medical University between January 2021 and December 2024. Patients were grouped based on immunotherapy received: tacrolimus (TAC) or glucocorticoids (GC). Peripheral blood samples were assessed for T lymphocyte subsets (CD3^+^, CD4^+^, CD8^+^) and B lymphocyte subsets (CD19^+^), alongside clinical parameters.

**Results:**

A total of 46 MG patients were included, with 23 patients in each treatment group. Baseline characteristics, including sex, age at onset, antibody profile, and thymic pathology, were comparable between the two groups (all P > 0.05), except for a significantly higher proportion of generalized MG in the TAC group (P = 0.017). Following treatment, the TAC group demonstrated a significantly lower absolute count of CD3^+^CD4^+^ T cells compared to the GC group (663.4 ± 345.5 × 10^6^/L vs. 952.5 ± 513.9 × 10^6^/L, P = 0.030). Additionally, the percentage of peripheral B cells in the tacrolimus group decreased significantly after treatment (from 11.8 ± 4.7% to 9.4 ± 4.4%, P = 0.006). In contrast, patients treated with glucocorticoids showed significant post-treatment increases in the absolute counts of CD3^+^, CD3^+^CD4^+^, and CD3^+^CD8^+^ T cells (all P = 0.001).

**Conclusion:**

Compared with patients receiving glucocorticoid therapy, those treated with tacrolimus exhibited significantly lower levels of peripheral CD3^+^CD4^+^ T cells after treatment. These findings provide insight into the differential immunomodulatory effects of these therapies in MG.

## Introduction

The AChR antibody is the main pathogenic antibody of myasthenia gravis (MG), which stems from AChR-specific B lymphocytes. The maturation of B lymphocytes requires the assistance of T lymphocytes (1–3). Glucocorticoids are the first-line treatment for MG, with clinical efficacy reported in 70–80% of cases. Tacrolimus (TAC), also known as FK506, is a macrolide drug isolated from Streptomyces. It is a calcineurin inhibitor that binds to calcineurin within T lymphocytes, thereby blocking the activation of the IL-2 gene signal transduction pathway. By influencing the activation and proliferation of T lymphocytes, it reduces the transformation of B lymphocytes into plasma cells, ultimately leading to a decrease in the production of pathogenic antibodies (4). TAC is currently widely used in the treatment of MG (5, 6). In addition, TAC has shown favorable efficacy and safety in both juvenile MG (JMG) and MuSK-MG (7, 8). Once the condition of MG stabilizes, the dosage of medication can be gradually decreased (9).

The immunopathogenesis of MG is closely linked to dysregulated lymphocyte function. The lymphocyte differentiation lineage encompasses T lymphocytes, B lymphocytes, natural killer (NK) cells, NKT cells, and ILC cells. Lymphocytes are a significant component of the immune system, accounting for 20 to 50% of the total white blood cell count in the peripheral blood. Among them, lymphocytes involved in adaptive immune responses mainly consist of αβT cells and B cells.

T cells mature in the thymus and then migrate to peripheral lymphoid tissues as naive cells. Upon encountering specific antigens, they activate, proliferate, and differentiate mainly into effector T cells, which perform immune functions such as cytokine production and target cell killing. A smaller portion becomes memory T cells, which persist long-term and enable faster responses upon re-exposure to the antigen. This process is the basis of cellular immunity. B cells mature in the bone marrow and migrate to peripheral lymphoid tissues. After antigen stimulation, they differentiate into plasma cells that secrete antibodies, mediating humoral immunity. Like T cells, a subset of B cells becomes memory B cells, providing long-lasting immune protection. Thus, both T and B lymphocytes play indispensable roles in MG pathogenesis and treatment responses. Given its mechanism of action, TAC not only reduces the generation of pathogenic antibodies but also carries the potential risk of infection, which may aggravate MG symptoms. Nevertheless, it might raise the risk of infection, and infection could further exacerbate the condition of MG.

Previous studies have explored the immunomodulatory effects of tacrolimus and glucocorticoids on lymphocyte subsets in autoimmune diseases, including MG. Tacrolimus primarily suppresses T cell activation and indirectly modulates B cell function, while glucocorticoids exert broader immunosuppressive effects by altering lymphocyte distribution and function (10–13). However, comparative immunophenotyping data in MG patients remain limited, and such data are increasingly important to guide individualized immunosuppressive regimens. Although direct comparisons between tacrolimus and glucocorticoids on peripheral lymphocyte subsets in MG are limited, emerging evidence suggests these drugs differentially affect immune cell profiles, which may inform individualized treatment strategies (14, 15). Therefore, the aim of this study is to compare the effects of tacrolimus and glucocorticoids on peripheral blood T and B lymphocyte subsets in patients with MG, with the goal of providing evidence-based guidance for treatment selection.

## Materials and methods

### Study design and participants

This retrospective case-control study utilized data from the Myasthenia Gravis Registry cohort at the First Affiliated Hospital of Fujian Medical University, covering the period from January 2021 to December 2024. The study was approved by the Ethics Committee of the First Affiliated Hospital of Fujian Medical University for Medical Research and Clinical Technology Application (Approval ID: [2020]243). Written informed consent was obtained from all participants enrolled in our institutional MG registry cohort.

Inclusion criteria were as follows (1): a clinical diagnosis of MG characterized by fluctuating, fatigable weakness in voluntary muscles, along with at least one of the following diagnostic confirmations: a. positive serum anti-acetylcholine receptor antibody (anti-AChR); b. a decremental response exceeding 10% in compound muscle action potentials during repetitive nerve stimulation at 3–5 Hz; or c. a definitive positive response to the neostigmine test (2); continuous treatment with either glucocorticoids or tacrolimus for a minimum of three months without interruption; and (3) available test results for peripheral blood T and B lymphocyte subsets both prior to and following treatment, with no instances of drug discontinuation between the two testing time points.

Exclusion criteria included (1): unplanned dose reduction or treatment discontinuation during the study period (2); administration of other immunosuppressive agents or targeted therapies or undergoing plasma exchange or intravenous immunoglobulin treatment during follow-up (3); incomplete testing for serum anti-AChR, anti-MuSK, and anti-LRP4 antibodies (4); a time interval of less than three months between the two lymphocyte subset evaluations; and (5) untreated thymoma patients were excluded to avoid potential confounding effects on lymphocyte subset measurements.

### Treatments and data collection

Patients were categorized into either the tacrolimus (TAC) group or the glucocorticoid (GC) group according to the type of immunotherapy received. Some patients in the TAC group received low-dose prednisone concurrently. Patients in the GC group received oral prednisone at an initial dose of 1–1.5 mg/kg/day once daily. The dosage was gradually tapered following clinical improvement and maintained at a low-dose regimen for long-term management.

Patients in the TAC group were administered oral tacrolimus at a dose of 1 - 1.5 mg twice daily (bid). The dosage was subsequently adjusted based on individual symptom response and blood tacrolimus trough concentration levels to achieve optimal therapeutic effect.

Clinical information was retrieved from the electronic medical record system via the Yidu Cloud Research Collaboration Platform. The collected variables included sex, age, age at disease onset, disease duration, affected muscle groups, thymus pathology, antibody profiles, treatment regimen, Post-Intervention Status (PIS), and adverse reactions. PIS refers to the Myasthenia Gravis Foundation of America (MGFA) Postintervention Status, a standardized clinical outcome measure used to evaluate treatment response in MG patients, as defined by Jaretzki et al. (16).

Reported adverse effects of glucocorticoid therapy included infection, hyperphagia, weight gain, central obesity, hypertension, hyperglycemia, cataracts, glaucoma, endocrine disturbances, psychiatric symptoms, osteoporosis, femoral head necrosis, and gastrointestinal symptoms. Tacrolimus-related adverse reactions encompassed hyperglycemia, infection, anemia, tremors, hypomagnesemia, hyperkalemia, hepatic and renal dysfunction, and, in rare cases, bone marrow suppression. Peripheral blood T and B lymphocyte subsets were analyzed by flow cytometry. The immunophenotypic data included the absolute count and percentage of CD3^+^ T cells, CD3^+^CD4^+^ T cells, and CD3^+^CD8^+^ T cells; the CD4^+^/CD8^+^ T cell ratio; and the percentage of CD45^+^CD19+ B cells. Patients with an onset age below 50 years were defined as having “early-onset MG,” while those with onset at 50 years or older were classified as “late-onset MG,” in accordance with established criteria (17).

### Statistical analysis

Statistical analyses were performed using SPSS version 27.0 (IBM Corp., USA), and data visualization was conducted with GraphPad Prism version 9.0 (GraphPad Software, USA). Continuous variables with normal distribution were expressed as mean ± standard deviation (SD), and group comparisons were conducted using independent samples t-tests or paired t-tests as appropriate. Non-normally distributed continuous data were presented as median (range) and analyzed using the Mann–Whitney U test or Wilcoxon signed-rank test. Categorical variables were expressed as counts and percentages (n, %) and compared using Fisher’s exact test.

## Results

### Clinical characteristics of included patients

A total of 46 patients were included in the analysis, with 23 patients in each group. The proportion of patients with generalized myasthenia gravis (GMG) was significantly higher in the TAC group compared to the GC group (73.91% vs. 39.13%, P = 0.017). No statistically significant differences were observed between the two groups regarding gender distribution, age at onset, antibody status, thymoma, or age at enrollment (all P>0.05). Detailed clinical characteristics are presented in **Table 1**.

**Table 1:** Baseline clinical characteristics of patients with MG.

Clinical Information	Total (n=46)	TAC (n=23)	GC (n=23)	P
Gender, number (%)				>0.999
Male	20 (43.48)	10 (43.49)	10 (43.49)	
female	26 (56.52)	13 (56.52)	13 (56.52)	
Age of onset (years)	45.74 ± 16.16	48.48 ± 17.61	43.00 ± 14.45	0.255
Age of onset type, number (%)				0.375
Early-onset MG	25 (54.35)	11 (47.83)	14 (60.87)	
Late-onset MG	21 (45.65)	12 (52.17)	9 (39.13)	
Type of MG, number (%)				0.017
OMG	20 (43.48)	6 (26.09)	14 (60.87)	
GMG	26 (56.52)	17 (73.91)	9 (39.13)	
Antibody, number (%)				0.681
AChR-MG	39 (84.78)	20 (86.96)	19 (82.61)	
Antibody-negative MG	7 (15.22)	3 (13.04)	4 (17.39)	
Thymoma, number (%)				0.71
Thymoma	9 (19.57)	5 (21.74)	4 (17.39)	
without thymoma	37 (80.43)	18 (78.26)	19 (82.61)	
Age at first test (years)	47.74 ± 14.72	51.17 ± 15.11	44.30 ± 13.81	0.393

TAC, tacrolimus; GC, glucocorticoid; MG, myasthenia gravis; OMG, ocular MG; GMG, generalized MG; AChR, acetylcholine receptor.

### Prior immunosuppressive therapy

Before the first lymphocyte subset assessment, 11 of 23 patients in the TAC group had previously received glucocorticoid therapy, with prednisone doses ranging from 10 to 60 mg/day. In the GC group, 2 of 23 patients had prior glucocorticoid exposure (prednisone 7.5 mg/day and 20 mg/day, respectively, with a treatment duration of less than one month). No patients in either group had received prior non-glucocorticoid immunosuppressive therapy (**Supplementary Table S1**).

### Treatment regimens

All patients received continuous immunosuppressive therapy with either tacrolimus or glucocorticoids during the interval between the two lymphocyte subset assessments. In the TAC group, the mean duration of tacrolimus treatment was 12.22 ± 7.32 months, whereas the GC group had a significant shorter mean treatment duration of 7.83 ± 4.01 months (P = 0.017). At the time of the final assessment, 17 patients in the TAC group were also receiving glucocorticoids, with a mean daily prednisone-equivalent dose of 9 ± 7.5 mg. In comparison, patients in the GC group had a significantly higher mean daily dose of 16.8 ± 7 mg (P < 0.001).

### Comparative analysis of lymphocyte subset between the TAC and GC groups

**Figure 1:**
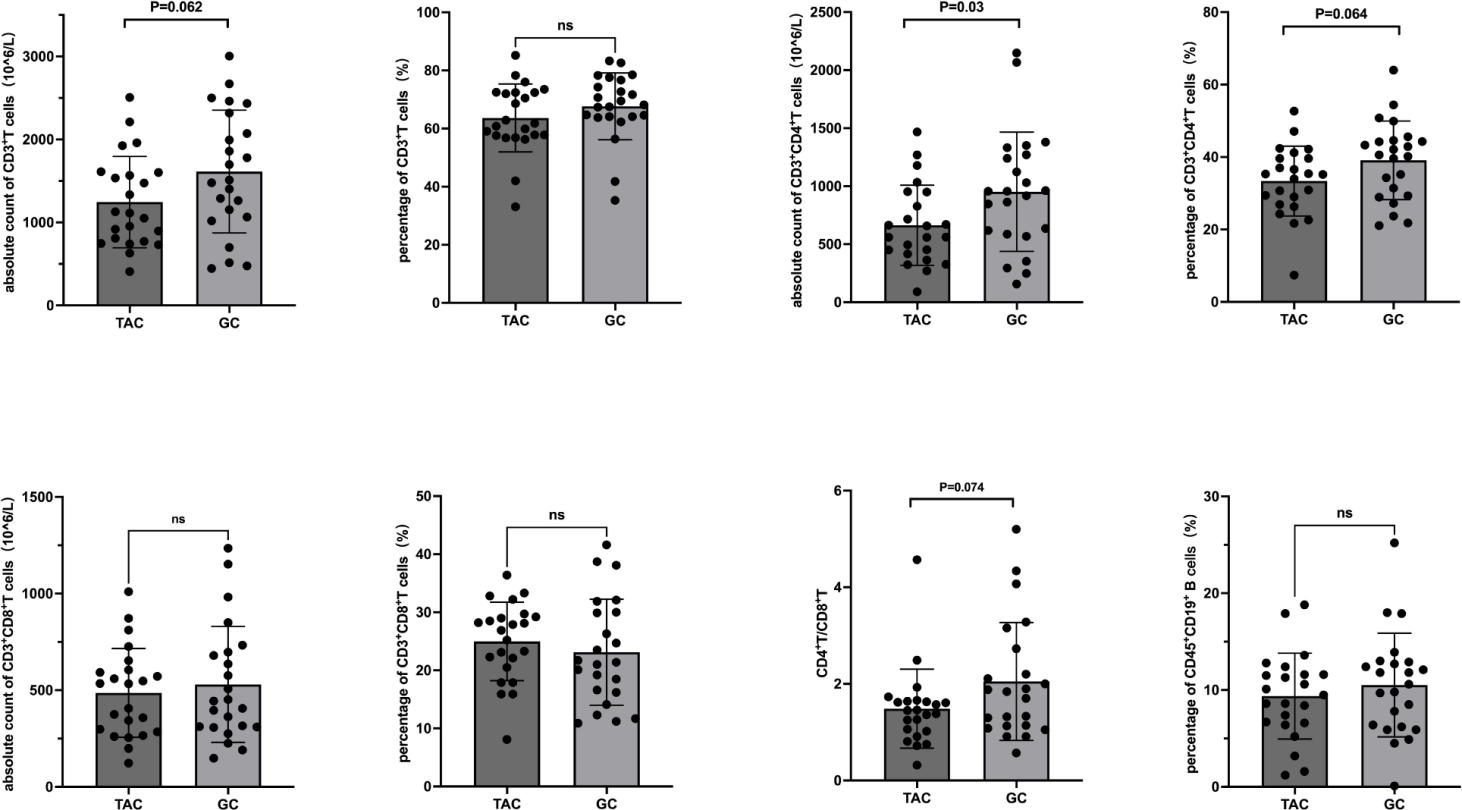
T and B Lymphocyte Subsets Following Treatment in the TAC and GC Groups. Left dark gray bars represent the TAC (tacrolimus) group, and right light gray bars represent the GC (glucocorticoid) group. TAC, tacrolimus; GC, glucocorticoid; CD, cluster of differentiation.

Prior to treatment, there were no statistically significant differences between the TAC and GC groups in the absolute counts or percentages of CD3^+^ T cells, CD3^+^CD4^+^ T cells, CD3^+^CD8^+^ T cells, CD4^+^/CD8^+^ ratios, or B cell percentages. Following treatment, the absolute count of CD3^+^CD4^+^ T cells was significantly lower in the TAC group compared to the GC group (663.4 ± 345.5 × 10^6^/L vs. 952.5 ± 513.9 × 10^6^/L, P = 0.030). Detailed lymphocyte subset data are presented in **Table 2** and illustrated in **Figure 1**.

**Table 2:** Peripheral blood lymphocyte subsets pre- and post- treatment in TAC and GC groups.

Classification	TAC	GC	P
Pre-treatment			
Absolute count of CD3+T cells (10^6/L)	1022 (287–3213)	969 (426–1740)	0.288
Percentage of CD3+T cells (%)	66.3 ± 7.3	65.6 ± 8.9	0.772
Absolute count of CD3+CD4+T cells (10^6/L)	637.7 ± 382.9	520 (238-1390)	0.660
Percentage of CD3+CD4+T cells (%)	36.4 ± 7.5	39.2 ± 8.5	0.245
Absolute count of CD3+CD8+T cells (10^6/L)	327 (110-1477)	311.5 ± 134.8	0.082
Percentage of CD3+CD8+T cells (%)	25.4 ± 8.6	21.4 ± 8.3	0.121
CD4+T/CD8+T	1.69 ± 0.99	2.16 ± 1.08	0.132
Percentage of B cells (%)	11.8 ± 4.7	12.1 ± 3.8	0.783
Post-treatment			
Absolute count of CD3+T cells (10^6/L)	1244.8 ± 549.9	1613.5 ± 740	0.062
Percentage of CD3+T cells (%)	63.7 ± 11.7	68.2 (35.3-83.3)	0.249
Absolute count of CD3+CD4+T cells (10^6/L)	663.4 ± 345.5	952.5 ± 513.9	0.030
Percentage of CD3+CD4+T cells (%)	33.4 ± 9.6	39.1 ± 10.8	0.064
Absolute count of CD3+CD8+T cells (10^6/L)	486.4 ± 229.5	530.2 ± 300	0.581
Percentage of CD3+CD8+T cells (%)	25.0 ± 6.8	23.1 ± 9.1	0.438
CD4+T/CD8+T	1.45 (0.32-4.57)	1.84 (0.57-5.2)	0.074
Percentage of B cells (%)	9.4 ± 4.4	10.5 ± 5.4	0.433

TAC, tacrolimus; GC, glucocorticoid; T cells, T lymphocytes; CD, cluster of differentiation; B cells, B lymphocytes.

### Pre- and post-treatment comparison in TAC group

**Figure 2:**
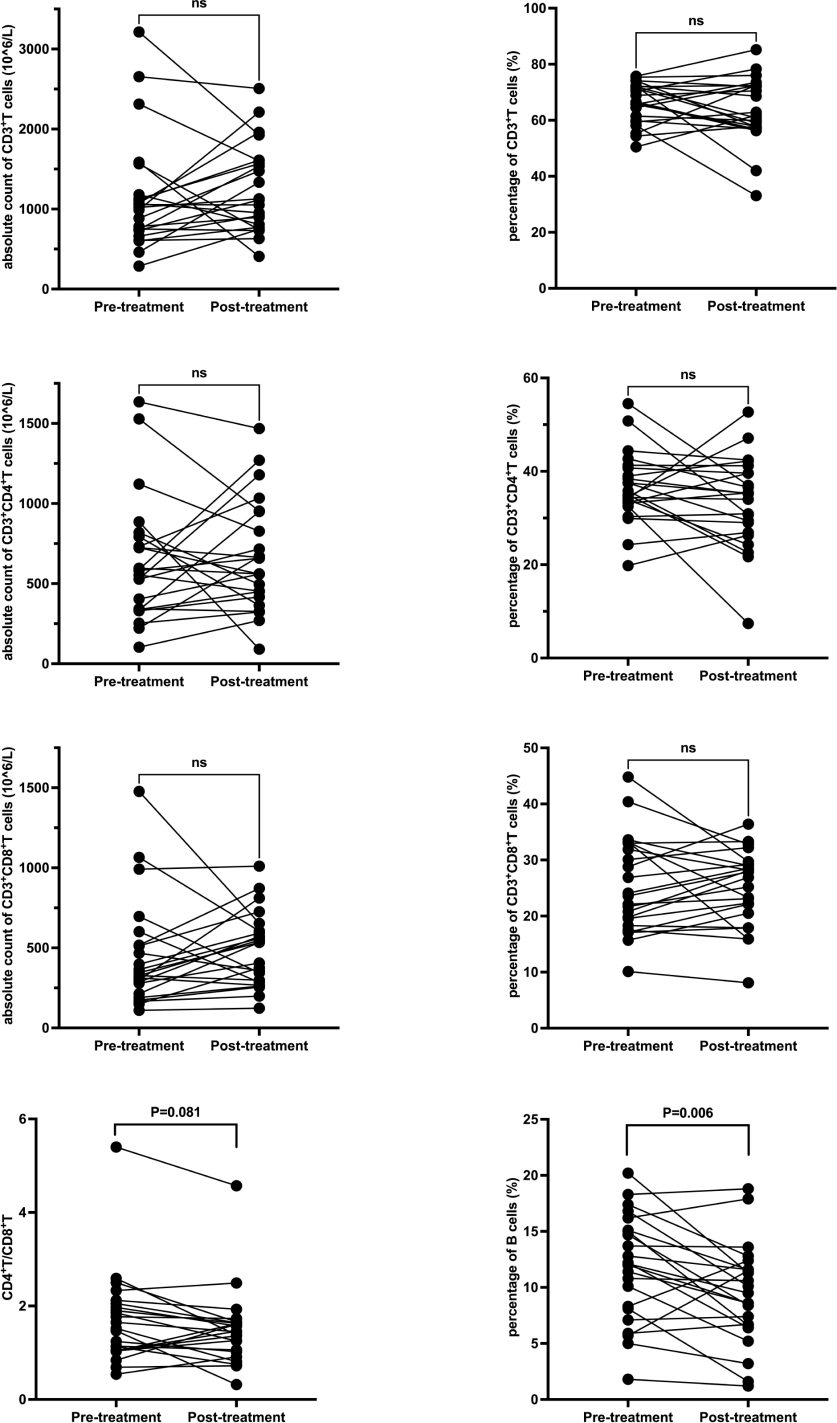
T and B Lymphocyte Subsets Following Tacrolimus Treatment. Each line represents an individual patient. CD, cluster of differentiation.

Within the TAC group, the percentage of B cells significantly decreased from 11.8 ± 4.7% to 9.4 ± 4.4% post-treatment (P = 0.006). No significant changes were noted in other lymphocyte subsets. Seventeen patients in the TAC group were receiving low-dose prednisone concurrently during the study period. Detailed data are presented in **Table 3** and **Figure 2**. In the subset of 17 patients in the TAC group who were concurrently receiving low-dose prednisone, the percentage of B cells significantly decreased from 11.5 ± 4.8% at baseline to 8.6 ± 4.3% after treatment (P = 0.003). No significant changes were observed in other lymphocyte subsets.

**Table 3:** Peripheral blood lymphocyte subsets pre- and post- treatment in the TAC group.

Classification	Pre-treatment	Post-treatment	P
Absolute count of CD3+T cells (10^6/L)	1022 (287-3213)	1244.8 ± 549.9	0.492
Percentage of CD3+T cells (%)	66.3 ± 7.3	63.7 ± 11.7	0.277
Absolute count of CD3+CD4+T cells (10^6/L)	637.7 ± 382.9	663.4 ± 345.5	0.745
Percentage of CD3+CD4+T cells (%)	36.4 ± 7.5	33.4 ± 9.6	0.165
Absolute count of CD3+CD8+T cells (10^6/L)	327 (110-1477)	486.4 ± 229.5	0.527
Percentage of CD3+CD8+T cells (%)	25.4 ± 8.6	25.0 ± 6.8	0.783
CD4+T/CD8+T	1.69 ± 0.99	1.45 (0.32-4.57)	0.081
Percentage of B cells (%)	11.8 ± 4.7	9.4 ± 4.4	0.006

TAC, tacrolimus; T cells, T lymphocytes; CD, cluster of differentiation; B cells, B lymphocytes.

### Pre- and post-treatment comparison in GC group

**Figure 3:**
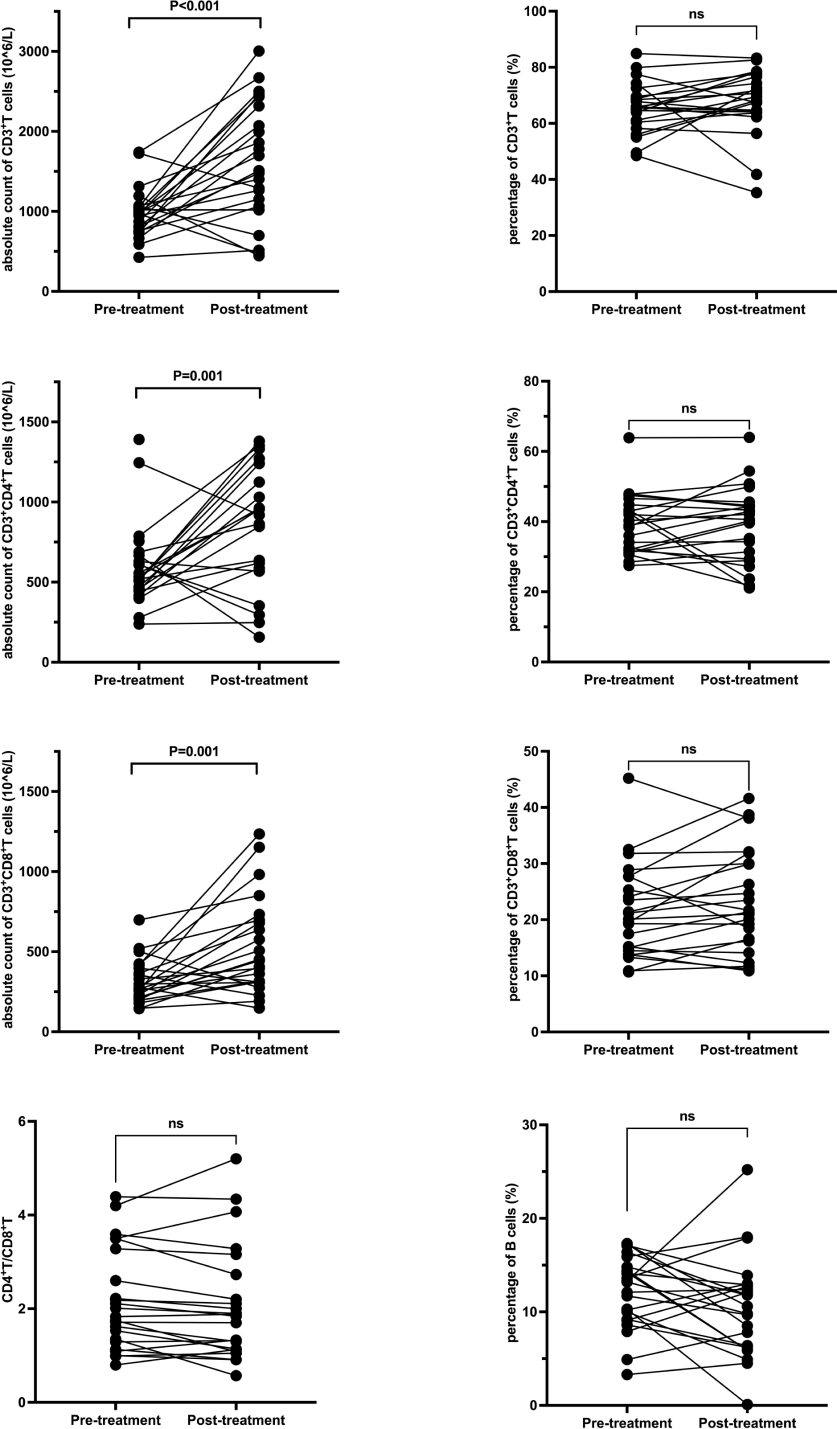
T and B Lymphocyte Subsets Following Glucocorticoid Treatment. Each line represents an individual patient. CD, cluster of differentiation.

In the GC group, post-treatment analysis revealed significant increases in the absolute counts of CD3^+^ T cells (from 969 × 10^6^/L to 1613.5 ± 740 × 10^6^/L, P < 0.001), CD3^+^CD4^+^ T cells (from 520 × 10^6^/L to 952.5 ± 513.9 × 10^6^/L, P = 0.001), and CD3^+^CD8^+^ T cells (from 311.5 ± 134.8 × 10^6^/L to 530.2 ± 300 × 10^6^/L, P = 0.001). No significant changes were noted in the percentages of these subsets or in the CD4+/CD8+ ratio. Detailed data are presented in **Table 4** and **Figure 3**.

**Table 4:** Peripheral blood lymphocyte subsets before and after treatment in the GC group.

Classification	Pre-treatment	Post-treatment	P
Absolute count of CD3+T cells (10^6/L)	969 (426-1740)	1613.5 ± 740	<0.001
Percentage of CD3+T cells (%)	65.6 ± 8.9	68.2 (35.3-83.3)	0.394
Absolute count of CD3+CD4+T cells (10^6/L)	520 (238-1390)	952.5 ± 513.9	0.001
Percentage of CD3+CD4+T cells (%)	39.2 ± 8.5	39.1 ± 10.8	0.943
Absolute count of CD3+CD8+T cells (10^6/L)	311.5 ± 134.8	530.2 ± 300	0.001
Percentage of CD3+CD8+T cells (%)	21.4 ± 8.3	23.1 ± 9.1	0.138
CD4^+^T/CD8^+^T	2.16 ± 1.08	1.84 (0.57-5.2)	0.207
Percentage of B cells (%)	12.1 ± 3.8	10.5 ± 5.4	0.162

GC, glucocorticoid; T cells, T lymphocytes; CD, cluster of differentiation; B cells, B lymphocytes.

### PIS and adverse events

At the time of the initial lymphocyte subset assessment, 13 patients in the TAC group were classified as being in the phase of disease onset, exacerbation, or worsening according to the MGFA PIS; 1 patient was unchanged, and 9 patients were improved. At the final assessment, 1 patient was in exacerbation, 1 was unchanged, 9 were improved, and 12 achieved minimal manifestation status. During the follow-up period, 8 patients in the TAC group experienced clinical worsening, and 4 developed infections, including pulmonary cryptococcosis (n = 1), bacterial pneumonia (n = 1), herpes zoster (n = 1), and COVID-19 (n = 1). In the GC group, 22 patients were in the phase of disease onset, exacerbation, or worsening at the time of the first lymphocyte subset assessment, while 1 patient showed improvement. By the final assessment, 1 patient remained in exacerbation, 1 had worsened, 10 showed improvement, 8 achieved minimal manifestation status, and 3 reached pharmacologic remission. During follow-up, 5 patients experienced clinical worsening, and no infections were recorded in this group (**Table 5**).

**Table 5:** Comparison of PIS status and adverse events between the two groups.

PIS	TAC (n = 23)	GC (n = 23)	P
PIS at first lymphocyte subset assessment			0.008
New-onset/Exacerbation/Worse	13 (56.5%)	22 (95.7%)	
Unchanged	1 (4.3%)	0 (0.0%)	
Improved	9 (39.1%)	1 (4.3%)	
PIS at final lymphocyte subset assessment			>0.999
Exacerbation/worse	1 (4.3%)	2 (8.7%)	
Unchanged	1 (4.3%)	0 (0.0%)	
Improved	9 (39.1%)	10 (43.5%)	
Minimal manifestation status/Pharmacologic remission	12 (52.2%)	11 (47.8%)	

## Discussion

This study investigated the changes in peripheral blood lymphocyte subsets in MG patients treated with tacrolimus compared to those treated with glucocorticoids. We found that tacrolimus treatment was associated with a significant decrease in the absolute count of CD3^+^CD4^+^ T cells, alongside downward trends in the absolute count of CD3^+^ T cells, the percentage of CD3^+^CD4^+^ T cells, and the CD4^+^/CD8^+^ T cell ratio. Additionally, the proportion of B cells significantly decreased following tacrolimus treatment. Conversely, glucocorticoid treatment resulted in a marked increase in the absolute counts of CD3^+^ T cells, CD3^+^CD4^+^ T cells, and CD3^+^CD8^+^ T cells. These findings indicate that tacrolimus and glucocorticoids exert distinct immunomodulatory effects on lymphocyte subsets in MG patients.

Tacrolimus primarily suppresses T cell activation by inhibiting interleukin-2 (IL-2) production, which leads to reduced proliferation of T lymphocytes, particularly helper CD4^+^ T cells. Our results align with previous studies showing tacrolimus’s inhibitory effect on CD4^+^ T cell proliferation, possibly mediated by suppression of dendritic cell function and enhancement of regulatory T cell (Treg) populations (18, 19). This selective downregulation of helper T cells likely contributes to tacrolimus’s immunosuppressive efficacy in MG. The significant reduction in peripheral B cell percentages observed after tacrolimus treatment suggests an indirect effect on B cells through modulation of T cell help, consistent with the drug’s known mechanism of blocking T cell-dependent B cell activation and differentiation into antibody-secreting cells (20).

Notably, our findings can be compared with previous work by Arslan et al. (21), who evaluated the effects of immunosuppressive therapies on follicular T helper and T helper 17 cells in MG. Their study reported correlations between these lymphocyte subsets and disease severity, emphasizing the impact of immunomodulatory agents on adaptive immune cell populations. By examining conventional T and B lymphocyte subsets in the context of tacrolimus and glucocorticoid therapy, our study complements these findings and extends the understanding of immunosuppressive effects in MG.

In contrast, glucocorticoids exert broad immunomodulatory effects by influencing lymphocyte differentiation, proliferation, apoptosis, and cytokine production. While glucocorticoids are generally thought to reduce lymphocyte counts through induction of apoptosis and inhibition of proliferation (22, 23), our data showed increased absolute counts of CD3^+^ T cells and subsets following moderate to low-dose glucocorticoid therapy. This discrepancy may be explained by the relatively low glucocorticoid doses used and the complex dual pro- and anti-apoptotic actions of glucocorticoids on different lymphocyte populations. Moreover, glucocorticoids can promote differentiation of naïve T cells into regulatory subsets, potentially contributing to immune regulation without necessarily reducing total lymphocyte numbers (24, 25).

Clinically, the decrease in CD4^+^ T cells and CD4^+^/CD8^+^ T cell ratio in the tacrolimus group correlated with a case of opportunistic infection, underscoring the importance of immune monitoring during treatment. This finding highlights the delicate balance between achieving immunosuppression and preserving host defense.

Despite these insights, several limitations warrant consideration. Most patients receiving tacrolimus were also treated with glucocorticoids, and prior glucocorticoid exposure may have influenced lymphocyte dynamics, complicating the isolation of tacrolimus-specific effects. Blood samples in the tacrolimus group were collected while patients were still on glucocorticoids, and data on tacrolimus monotherapy are not available, which limits the interpretation of tacrolimus-specific effects. Furthermore, the study did not differentiate between various CD4^+^ T cell subsets such as effector versus regulatory T cells, limiting detailed immunophenotypic interpretation. Lastly, B cell analysis was restricted to proportional changes without absolute counts, which may be affected by concurrent shifts in T cell populations.

## Conclusions

In conclusion, our data demonstrate that tacrolimus treatment in MG patients results in significant reductions in helper T cells and B cells, consistent with its mechanism of targeted immunosuppression. In contrast, glucocorticoids at moderate doses appear to increase T cell counts, reflecting their complex immunomodulatory effects. These findings provide valuable immunological insights that may inform personalized treatment strategies and highlight the need for careful immune monitoring to balance efficacy and infection risk.

## Data Availability

The original contributions presented in the study are included in the article/**Supplementary Material**. Further inquiries can be directed to the corresponding author.
