# Supplementary material for: Impacts of tacrolimus and glucocorticoids on peripheral blood T and B lymphocyte subsets in myasthenia gravis
Source: Front Immunol. 2025 Oct 15;16:1667799. doi: 10.3389/fimmu.2025.1667799 (PMC12568596; doi:10.3389/fimmu.2025.1667799)
Supplement: Supplementary file 1 [file Table1.docx]

Table S1. Prior immunosuppressive treatments before baseline testing

| **Treatment group** | **TAC (n=23)** | **GC (n=23)** |
| --- | --- | --- |
| **Prior non-glucocorticoid immunosuppressive therapy** | 12 | 21 |
| **Prior glucocorticoid immunosuppressive therapy** | 11 | 2 |
